# Progress in the Articulation of Undergraduate and Graduate Public Health?

**DOI:** 10.3389/fpubh.2015.00022

**Published:** 2015-02-10

**Authors:** Joel M. Lee, Leonard H. Friedman

**Affiliations:** ^1^College of Public Health, University of Georgia, Athens, GA, USA; ^2^Milken Institute School of Public Health, The George Washington University, Washington, DC, USA

**Keywords:** public health education, undergraduate, articulation, baccalaureate

Historically, in the absence of baccalaureate education in the public health, entry level education was offered at the graduate level in the Master of Public Health (MPH) degree. As MPH education was not preceded by baccalaureate education in the discipline, there are not typically prerequisite requirements for MPH admission, and introductory coursework in public health has always been offered at the graduate level. Other disciplines including business and nursing offer joint and dual degree programs; however, these programs are typically designed to accelerate completion of baccalaureate and graduate degrees (1–4) rather than supplement graduate with undergraduate education. While the developers of new undergraduate public health programs may look at the experience in other disciplines, public health presents the atypical characteristic that well-established graduate education preceded undergraduate education. In addition, review of a variety of accelerated programs at different universities demonstrates that they tend to be unique to individual universities rather than of a standard design.

The recent establishment of undergraduate public health degree programs presents an interesting situation for students seeking an MPH degree following completion of their baccalaureate degree. The typical curricula at both the undergraduate and graduate levels will require similar, but not identical introductory courses that likely vary in depth and breadth as detailed elsewhere by the authors (5, 6). Consequently, these students will potentially experience unintended duplication of content with additional costs of time and money.

As undergraduate public health education began to experience interest and rapid growth, one of the authors of this commentary published a paper *Articulation of Undergraduate and Graduate Education in Public Health* (7). The paper addressed the benefits of designing strategies to better coalesce or articulate undergraduate and graduate education, identifying barriers to articulation and strategies to achieve alignment between undergraduate and graduate education. The paper additionally presented a set of issues that were unanswered and require careful consideration. It is now 6 years later, and the authors wish to assess progress in articulating undergraduate and graduate public health education to achieve greater harmony between the two degrees. In doing so, we presume that improving the articulation of undergraduate and graduate programs to better align public health in a manner similar to many other disciplines is beneficial and have not heard arguments to the contrary.

While there has not been a systematic national effort to catalog implementation of these strategies, the authors have sought to identify examples of progress where they exist. Each of the proposed strategies and observations offering examples of current status follow.

- Plans for formally articulated programs might be established at a single university where both undergraduate and graduate public health education are offered, as a memorandum of agreement among two or more programs, SPHs, or universities; and/or a national policy supported by the Association of Schools and Programs in Public Health (ASPPH, formerly ASPH) and/or Council on Education in Public Health (CEPH): as an example of unified degrees in a single unit, Tulane University School of Public Health and Tropical Medicine offers a continuous study BSPH/MPH combined degree program where students may complete up to 12 graduate credits in public health core courses that may be applied to both the BSPH and the MPH. A cross-university option is offered by the Johns Hopkins University Bachelor of Arts/Master of Sciences in Public Health (BA/MSPH) programs as a coordinated academic collaboration between the Krieger School of Arts and Sciences and the Johns Hopkins Bloomberg School of Public Health. The option enables students to complete the two degrees in 5–6 years. Inter-institutional agreements are far more complicated than a single school of public health offering both degrees, or even two colleges at the same university. An example of a 5-year inter-institutional program is offered as a baccalaureate degree from Mount Mary College and an MPH degree from the Medical College of Wisconsin. In this model, students have the opportunity to complete up to 15 credits of graduate coursework that apply to both undergraduate and graduate degrees. Interestingly, the undergraduate degree may be in any discipline, and the College does not offer a free standing undergraduate degree in public health. Presently, a national model has not yet been proposed to address this type of initiative at a broader level.
- Graduate programs could base course waivers on detailed content analysis of undergraduate-course syllabi or by competency examinations using the ASPH MPH core competencies: while it is likely that course waivers are being accomplished on a case-by-case basis in various programs, there is little evidence of waivers being promoted as policy in graduate programs. Further, CEPH accreditation presently calls for a minimum requirement of 42 semester hours for the MPH degree and this does not offer the flexibility to reduce credit requirements based upon prior course competencies. Consequently, at best a waiver would enable a student to avoid duplication by completing advanced coursework, but not an accelerated degree.
- Early matriculation to graduate school might be offered prior to the awarding of a baccalaureate degree, where the baccalaureate degree requirement is either waived or awarded upon completion of the first year of graduate education. This model is most common with early admission to medical or dentistry schools: in contrast to professional degrees, most graduate programs require a baccalaureate degree for matriculation, and the innovation appears limited to professional degrees in the awarding of baccalaureate degrees. It does not appear that any accredited MPH programs currently offer this option.
- Graduate programs, with the approval of the graduate schools that govern their degrees, could permit current undergraduates to enroll in graduate courses applicable to graduate degrees: while there may be restriction associated with eligibility based upon total hours earned, grade point average to be eligible, and/or a maximum number of graduate courses that may be completed, the opportunity for undergraduates to enroll in graduate courses appears to be in place in many universities. However, in the absence of a formal process to apply this coursework to an MPH degree, it is less clear how completion of graduate work prior to entering a graduate program would benefit a student, and it could potentially complicate the pursuit of an MPH degree.
- Graduate programs could create undergraduate prerequisite courses for all entering students in areas such as overview of public health, introductory epidemiology, introductory biostatistics, public health biology, and/or ethics: the absence of specific prerequisites for matriculation into an MPH program does require offering introductory coursework for incoming students and limits the number of advanced courses that may be completed in a 2-year-curriculum. Requiring prerequisite courses for graduate education would better align with graduate education in other disciplines, but based upon the limited number of baccalaureate public health programs would currently reduce the eligible applicant pool for MPH admissions, as well as affecting non-traditional students returning to school. Further, while a prerequisite in an area such as biology or statistics might be more easily defined, the current absence of standardized undergraduate courses in public health may result in diversity in the preparation of students. The recent establishment of CEPH accreditation of freestanding baccalaureate programs may aid in standardizing these courses in the future. Presently, it does not appear that any accredited MPH currently specifies public health-related course prerequisites for admission.
- Graduate programs, with the approval of graduate schools, could grant academic credit for selected, previously completed undergraduate courses, reducing hours required for the articulated degrees: while the previously mentioned formally articulated programs fit this model, it does not appear that this option is being formally offered. However, it may be available on an *ad hoc* basis for individual students. (This would also require CEPH’s concurrence to revise the MPH 42 semester-hour accreditation requirement to accommodate undergraduate work.)
- Duplication of coursework could be avoided by waiving specific graduate-course requirements based upon undergraduate work, and substituting advanced graduate courses to achieve greater depth in curriculum content: in contrast to the articulated programs that use graduate-course work to meet undergraduate degree requirements, this strategy would call for application of baccalaureate coursework to graduate requirements. In addition to assessing adequacy of content in these courses, in universities where the MPH is located in the graduate school approval would be challenging. This would also require CEPH’s concurrence to revise the MPH 42-semester-hour accreditation requirement to accommodate undergraduate work, and consequently the strategy is not presently viable.
- Programs could waive required field experience requirements for students with baccalaureate practicum experiences admitted directly to graduate education: in addressing “practical skills” CEPH accreditation criteria call for a planned, supervised and evaluated practice experience for all graduate professional degree students. However, individual waivers may be granted based on well-defined criteria, the possession of a prior professional degree in another field, or prior work experience. It is possible that an MPH student with an undergraduate degree in public health may be eligible for a waiver; however, the value added in an additional practical experience would need to be carefully considered.
- A small number of advanced or specialty MPH or MSPH degree programs designed exclusively for graduates of baccalaureate public health curricula could be established: this remains an interesting strategy and would be reflective of education in a discipline such as nursing. At this stage of baccalaureate development it is unlikely that such an MPH program could generate the critical mass of applicants to be successful, while concurrently denying access to other applicants.
- Students with a baccalaureate degree in public health could be offered the opportunity to bypass a master’s degree and proceed directly to doctoral education: this option exists in some universities for disciplines in the arts and sciences. More recently, early entry or “fast-track” options in doctoral education in nursing have grown for baccalaureate graduates. While atypical, admission criteria for the Doctor of Public Health degree in Public Health Policy and Management at the University of Arizona Mel and Enid Zuckerman College of Public Health provide that applicants with a bachelor’s degree and 5 years of public health work-related experience may be admitted into the program. However, these students are required to complete the five core MPH courses in addition to the minimum DrPH credit hours of course work in the major. It is unlikely that this model will see rapid growth.

The examples presented are anecdotal as there presently is not a formal national mechanism to collect and disseminate information about undergraduate program innovations. Presently, there is not even agreement in regard to the definition or number of undergraduate programs (Tarasenko and Lee, submitted). While the concept of articulation of the MPH with other graduate and professional degrees including the MSW, MA, MS, MHA, MBA, MD, PharmD, DMD, MSN, and JD is well accepted, articulation with undergraduate degrees is a relatively new framework. Issues related to course academic content and rigor, degree requirements, graduate school policies, and accreditation must be considered in planning for the articulation of undergraduate and graduate public health degrees. Efforts by ASPPH, CEPH, and the Association of American Colleges and Universities have contributed to the advancement in the development of undergraduate education; however, issues related to national policies promoting degree articulation have not yet been addressed.

As both interest in undergraduate education and the number of programs and students grow, issues related to the career paths of baccalaureate graduates and their opportunities to pursue graduate degrees in an efficient manner will continue to receive attention. While examples of articulation better aligning undergraduate and graduate public health education are available, these examples tend to be exceptions to convention associated with a not yet mature undergraduate degree. Articulation has the potential to facilitate the admission of students into MPH programs and additionally, provide opportunities for MPH programs to adjust their curriculum to accommodate students with relevant educational preparation. Given the ever increasing direct and indirect costs of graduate education, schools and programs in public health might wish to consider creating opportunities for students to complete their degrees in a shorter period of time using articulation with undergraduate public health education as one way to accomplish this goal. Actions to address these policies will facilitate public health education and the students we serve.

## Author Note

The authors who presently serve in administrative leadership positions in graduate education have both previously served as directors of undergraduate programs in health administration as well as on committees and the Board of the Association of University Programs in Health Administration and as site visitors for Council on Accreditation in Health Management Education.

## Conflict of Interest Statement

The authors declare that the research was conducted in the absence of any commercial or financial relationships that could be construed as a potential conflict of interest.
